# Resections in London Hospitals

**Published:** 1868-01

**Authors:** 


Resections in London Hospitals.—Prof. Edmund Andrews, in a letter to the *Chicago Medical Examiner*, writes as follows:

"Resections of the knee are extensively practiced, even in children. The surgeons deny that it will prevent the limb from growing, provided you do not remove the whole of the epiphysis. The practice here is very often to amputate or resect inflamed knees, in cases where a Chicago surgeon would save the limb and effect a cure. They operate in the first stages, while the disease is yet a simple inflammation, without suppuration or caries; and while, according to American experience, the knee is perfectly curable. In justification of this practice, they say that the cure, if accomplished at all, would be excessively slow, and, therefore, the hospitals could not keep the patients long enough. They would go out and run about on their limbs, and exasperate the disease to actual caries; therefore they think it better to operate at once. The real fact is this: They treat their patients only by medicine, local applications, and rest in the foul air of the wards, and find that they die. As a general rule, they are grossly ignorant of the fact that adhesive-strap extension and pure air will cure the patient without operation. So they take the easiest course, and cut out the joint, or cut off the limb, and thus end the matter, and frequently kill the patient."

